# Informal caregiving and markers of adiposity in the UK Household Longitudinal Study

**DOI:** 10.1371/journal.pone.0200777

**Published:** 2018-07-19

**Authors:** Rebecca E. Lacey, Anne McMunn, Elizabeth Webb

**Affiliations:** Research Department of Epidemiology and Public Health, University College London, London, United Kingdom; Medical University of Vienna, AUSTRIA

## Abstract

**Objectives:**

The aim was to investigate associations between caregiving and adiposity using a representative UK longitudinal study. We also investigated whether associations differed by age, gender and caregiving characteristics.

**Methods:**

Data on 9,421 participants aged 16+ from three waves (2009–2012) of the UK Household Longitudinal Study were used. Body mass index, waist circumference and percentage body fat were assessed. Caregiving and caregiving characteristics (hours per week, number of people cared for, co-resident caregiving and combining working and caregiving) was available from the prior wave. Gender-stratified associations between caregiving/caregiving characteristics with adiposity were tested. Covariates included caregiver’s health, socioeconomic position, parenthood and partnerships.

**Results:**

Caregiving was associated with higher adiposity for women but not men. Younger women caregivers had particularly higher levels of adiposity. Men combining part-time paid work with caregiving had higher levels of adiposity than men working full-time and not caregiving. Women aged 16–44 or 65+ had particularly high levels of adiposity when combining full-time work and caregiving, compared to full-time work alone.

**Conclusion:**

The health of caregivers should be a public health priority, particularly for younger women and those combining paid work with caregiving responsibilities.

## Introduction

There are approximately seven million informal caregivers in the United Kingdom (UK) and an estimated 20% of the adult population in the United States providing informal care, 60% of whom are women in both countries [1,2]. Informal caregiving has large economic importance; the value of care in the United States in 2009 was estimated at $450 billion, much larger than the $361 billion spent on Medicare that year [3]. Similarly carers have been estimated to save the UK economy £132 billion per year [1]. Informal caregiving is increasing over time in response to rising life expectancy [4] and adult social care funding cuts [5].

Meta-analyses conclude that, on average, caregiving is associated with poorer psychological and physical health [6–8]. However the caregiving research has largely utilised cross-sectional studies, samples of older adults or focussed on caregivers to specific patient groups, such as dementia or cancer sufferers [8]. Caregiving is linked to poorer health through the uptake of risky health behaviours, physical strain and fatigue, reduction in social support, loss of self-identity, change in the nature and reciprocity of the caregiver-care recipient relationship (particularly in the case of caring for someone with dementia), caregiver burden, psychological distress, conflict between caregiving and work responsibilities, and financial burden, for example due to reduced hours of paid work [9–14]. These mechanisms may accompany, and feed into, a physiological stress mechanism through hypothalamic-pituitary-adrenal axis dysregulation. Previous research has shown that caregivers have higher salivary cortisol compared to non-caregivers [15]. One manifestation of this stress mechanism is increased fat deposition [16]. Vitaliano and colleagues [12] investigated the association between caregiving for a spouse with Alzheimer’s Disease and body mass index (BMI) in the United States. Caregivers experienced an increase in BMI, partly explained by calorific intake and perceived control. Also Hajek and Konig [17] explored the association between informal caregiving and change in BMI over five waves of the German Aging Survey (adults aged 40 years and over), finding that BMI increased when men initiated caregiving but not women. The association between caregiving and adiposity is yet to be examined in a broader population of the UK population aged 16+ and not restricted to caregivers providing care for people with specific health conditions, such as dementia.

Associations between informal caregiving and health depend upon several factors. Firstly, the caregiving intensity shows graded relationships with health. For example, UK data showed that caregivers providing fewer than 10 hours per week of care did not have worse health than non-caregivers [18,19], and in one study was associated with lower mortality [20]. Combining caregiving with work presents additional challenges and this combination is becoming increasingly common with extended working lives [21]. Unlike other social roles, caregiving is relatively inflexible, often requiring tasks to be completed at specific times [22]. This social role combination is likely to have health implications. For instance, caregivers who were employed had poorer self-rated health [23]. This has yet to be investigated in relation to an objective measure of health, such as adiposity. Gender is also important. Women are more likely to be caregivers from early adulthood to early old age [24], but men are more likely to be caregivers in older age [25]. Women caregivers are more likely to report poorer health than male caregivers [6], often because they have more intense caregiving responsibilities [26] and consequently report higher caregiver burden and stress [27]. Women caregivers are also more likely to reduce their working hours or leave paid work for caregiving activities, and this is further linked to higher caregiving intensity [21]. Finally, age is important but previous findings have been inconsistent in this respect. There is a suggestion that older caregivers have poorer health due to less social interaction outside of the caregiver-care recipient relationship, less use of formal support systems and reduced financial, psychological and physical resources [8]. One meta-analysis [8] found that older caregivers had worse physical health than younger caregivers. However the authors also found that younger caregivers reported more depression and caregiver burden, possibly because of the competing demands of caregiving with work and family. Further research is required which examines the importance of age.

The aim of the present study was to assess whether caregiving was associated with higher adiposity in a nationally representative, longitudinal sample of people aged 16+. We also investigated whether caregiving characteristics, such as caregiving hours, the number of people cared for, whether caregiving was combined with paid work, exhibited graded associations with adiposity, and whether associations between caregiving and adiposity were modified by age.

## Methods

### Data

This study used data from the UK Household Longitudinal Study (UKHLS), a large nationally representative sample of 40,000 UK households initiated in 2009 [28]. The UKHLS has a panel design with a stratified, clustered, equal probability sample. The study incorporates participants of the British Household Panel Study initiated in 1991. All adults aged 16+ in each household are surveyed every year. A health assessment was performed in 2010–2012 (waves 2 and 3) for a sub-sample of participants who resided in Great Britain (England, Scotland and Wales) and who had completed a full interview in English at the previous wave (n = 20,644, 15,591 of whom were UKHLS participants (wave 2) and 5,053 were originally British Household Panel Study participants (wave 3)).[29] This assessment included measures of adiposity (described further below) and so this study used data from waves 1–3 (2008–2012).

### Ethics statement

Written informed consent was obtained from all study participants for all waves. Ethical approval was obtained from the University of Essex ethics Committee.

### Measures

#### Caregiving and caregiving characteristics

All participants were asked at each survey whether they looked after anyone who was sick, disabled or elderly either within or outside of the household. This information was used to indicate whether participants were caregivers or non-caregivers at waves 1 and 2. Caregivers were subsequently asked to report the number of hours per week spent caregiving. Caregiving hours were categorised as not caregiving, 1–9, 10–19, or 20+ hours per week. These categories were based on existing literature and the available banded caregiving hours available. Additional caregiving variables included the number of people being cared for (categorised as not caregiving, one person or two or more people), whether the caregiver was caregiving for someone inside the household (co-resident caregiving) or just outside of the household, and the combination of caregiving with paid work (not working or caregiving, caregiving but not working, caregiving with part-time work (≤30 hours/week), caregiving with full-time work (>30 hours/week), working part-time but not caregiving and working full-time but not caregiving).

#### Adiposity

Three adiposity measures were available from the nurse visit–BMI, waist circumference and percentage body fat. BMI was calculated using measured height and weight as weight(kg)/height(metres)^2^. Waist circumference was measured three times by a study nurse and the mean across all three was used. Percentage body fat was measured using bioelectrical impedance using a digital floor scale on a hard floor surface. 19,872 participants had a valid BMI measure, 20,294 had a valid waist measurement and 18,808 had a valid body fat measurement.

#### Covariates

Covariates were gender, banded age (16–44, 45–64, or 65+ years), presence of a longstanding illness or disability, educational attainment (the highest qualification attained: no qualifications, General Certificate of Secondary Education/Ordinary-level qualifications or equivalent, Advanced-level qualification or equivalent, or degree or higher qualification), social class (National Statistics Socio-Economic Classification (NS-SEC) of current or last job: management and professional occupation, intermediate occupation or routine occupation/never worked), partnership status (single, married/civil partnership and living with a partner, separated or divorced, widowed, or cohabiting), number of dependent children aged 18 or under, net equivalised household income per month (quintiles), and smoking status (never-, ex- or current smoker). Household income was chosen over individual income, as it is more likely to reflect available household resources for the purchasing of formal care. By equivalising household income we have taken into account household composition. All variables, with the exception of smoking status which was only available at wave 2/3 and adiposity, were used at the survey prior to the health assessment (wave 2 for the UKHLS sample component and wave 3 for the British Household Panel Study sample component, referred to hereafter as ‘t1’).

#### Missing data

Participants with complete data on caregiving, covariates and adiposity were included in the analyses. Table 1 shows the number of missing values on each variable of interest and how the complete case sample differed from those with observed data. This exploration shows that our sample was less likely to be caregiving and were more socio-economically advantaged and younger than those with observed data. However complete cases had higher BMI and percentage body fat. The sample size for analyses with BMI was 9,190 (58.5% women), 9,318 (58.2% women) for waist circumference and 8,778 (59.1% women) for percentage body fat. The descriptive statistics shown in Table 1 are given for those with at least one adiposity measure who had information on caregiving and covariates (n = 9,421).

**Table 1:** Characteristics of the study sample.

	Men		Women					
	N	%	N	%	*P* gender difference	Distribution of excluded sample Mean/%	Number missing	*P* compared to whole sample
***Outcomes***								
BMI, mean (SD)	28.1	4.9	27.8	6.0	0.005	27.6	611	0.004
Waist, mean (SD)	99.2	13.3	88.6	14.1	<0.001	92.9	189	0.279
% body fat, mean (SD)	23.5	9.3	35.9	8.2	<0.001	29.7	1675	<0.001
***Caregiving***								
Caregiving								
No	3502	89.9	4637	85.7	<0.001	69.8	4650	<0.001
Yes	432	10.1	850	14.3		30.2		
Caregiving hours								
Not caregiving	3502	89.9	4637	85.7	<0.001	65.7	4217	<0.001
1–9 hours/week	345	8.1	585	9.1		16.8		
10–19 hours/week	43	1.1	139	1.8		5.3		
20+ hours/week	44	0.9	126	1.6		12.2		
Number of people cared for								
Not caregiving	3502	89.9	4637	85.7	<0.001	79.3	5249	<0.001
1 person	297	6.8	585	9.8		14.6		
2+ people	135	3.3	265	4.5		6.2		
Combining work with caregiving								
Not working or caregiving	1337	30.8	1911	34.6	<0.001	41.4	6015	<0.001
Caregiving but not working	185	3.9	349	5.9		21.8		
Caregiving with PT work	32	0.8	274	4.5		4.3		
Caregiving with FT work	215	5.4	227	3.9		6.9		
Working PT but not caregiving	239	6.7	1394	24.9		7.8		
Working FT but not caregiving	1926	52.4	1332	26.2		17.9		
Co-resident caregiving								<0.001
Not caregiving	3502	89.9	4637	85.7	<0.001	69.7	4642	
Non-resident caregiving	45	1.1	85	1.5		20.5		
Co-resident caregiving	387	9.0	765	12.7		9.8		
***Covariates***								
Age								
16-44yrs	1440	46.1	2424	50.2	<0.001	40.9	0	<0.001
45-64yrs	1449	33.3	2168	35.7		32.1		
65+yrs	1045	20.7	895	14.1		27.1		
NS-SEC								
Management & professional	1809	42.6	1978	33.8	<0.001	33.0	3972	<0.001
Intermediate	544	14.0	1213	21.6		30.8		
Routine & never worked	1581	43.5	2296	44.6		36.2		
Highest qualification								
No qualifications	464	11.1	714	12.6	<0.001	19.1	1106	<0.001
GCSE/equivalent	1151	29.7	1864	32.9		32.9		
A-level	848	22.6	906	18.2		19.5		
Degree/other higher	1471	36.7	2003	36.4		28.6		
Net household income quintiles								
Lowest	411	10.4	672	12.8	<0.001	21.9	1076	<0.001
2	687	17.9	1012	18.9		21.7		
3	820	21.7	1191	22.1		19.3		
4	976	24.2	1319	23.5		18.8		
Highest	1040	25.9	1293	22.7		18.3		
Longstanding illness or impairment								
No	2391	64.9	3477	65.2	0.769	58.7	1063	<0.001
Yes	1543	35.1	2010	34.8		41.3		
Partnership status								
Single	333	13.1	628	16.1	<0.001	28.1	1290	<0.001
Married & living with partner	2886	66.1	3519	58.9		38.5		
Separated/divorced	247	5.5	669	10.8		15.1		
Widowed	55	1.2	137	2.3		12.6		
Cohabiting	413	14.1	534	12.0		5.7		
Number of dependent children <18yrs, median (IQR)	0	0,0	0	0,1	<0.001	0	1284	<0.001
Smoking status								
Never smoker	1380	36.0	2487	45.5	<0.001	42.8	5008	0.277
Ex-smoker	1798	42.3	1939	34.1		36.1		
Current smoker	756	21.7	1061	20.4		21.1		

Abbreviations: A-level, Advanced level; BMI, body mass index; FT, full-time work; GCSE, General Certificate of Secondary Education; IQR, interquartile range; NS-SEC, National Statistics Socio Economic Classification; PT, part-time work; SD, standard deviation. Actual numbers and weighted percentages shown. Descriptive statistics shown for those with at least one adiposity measure with complete data on all analysis variables (n = 9,421)

### Statistical analyses

Linear regression was used to test associations between caregiving (binary) at t1 and BMI, waist circumference and percentage body fat at t2 (‘crude association’). A second model adjusted for age group (‘age-adjusted’). A final model was run including longstanding illness or disability, educational attainment, social class, partnership status, number of dependent children, household income and smoking status. In addition, age-caregiving interactions were tested throughout using a Wald test. Where interactions were present, estimates were stratified by age group. The same modelling strategy was applied to the analysis of caregiving characteristics, substituting each additional caregiving variable (caregiving hours, combining work and caregiving, number of people cared for and co-resident caregiving) in place of caregiving status. In order to minimise the problem of multiple comparisons, a Bonferroni corrected p value was applied based on three adiposity outcomes (0.05/3 = 0.0167). Analyses were gender-stratified, as women are more likely to be informal caregivers and to undertake more intensive caregiving responsibilities.[24] Consequently previous research has shown that the relationship between caregiving and health is generally stronger for women.[6] Also, the relationship between social exposures, such as social roles, and adiposity is generally stronger for women compared to men.[30] Survey weights were applied to account for the design, unequal probabilities of selection, differential non-response and potential sampling errors.

## Results

Women more commonly reported being a caregiver (14.3%) compared to men (10.1%) in our sample (Table 1). Women also provided more hours of caregiving per week; 14.8% of women caregivers were caring for >20 hours/week, compared to 10.2% of men, and men were more likely to report caregiving for <10 hours/week. In addition, women caregivers were more likely to be combining caregiving and paid work. Women were also more likely than men to be caring for more than one person and to have caregiving responsibilities within the household. Men had higher mean BMIs and waist circumferences compared to women (mean BMI, men: 28.1kg/m^2^, women: 27.8kg/m^2^; mean waist circumference, men: 99.2cm, women: 88.6cm). However women had higher percentage body fat than men (mean body fat, men: 23.5%, women: 35.9%). Men in the sample were more likely to be in managerial or professional occupations and were more likely to have A-level qualifications, although men and women were similar in the proportion who had at least a degree qualification. Men and women had similar levels of household income in our sample, however men were more likely to be partnered, and to be a current or ex-smoker.

### Caregiving and adiposity for men

Men who were informal caregivers had higher BMIs (1.07, 95% Confidence Intervals (CI): 0.33, 1.81), waist circumference (3.20cm, 95% CI: 1.62, 4.78) but not percentage body fat (1.14, 95% CI: -0.14, 2.41) in crude models (Table 2). However, once age was included these associations were no longer statistically significant. There was little variation in adiposity in relation to hours of caregiving, the number of people cared for or whether the participant was engaged in any co-resident caregiving. Compared to men who were working full-time but not caregiving, men who weren’t working but were caregiving had higher waist circumference, but this was no longer significant after then inclusion of age in the model. Similarly men combining caregiving with full-time work had lower BMIs but this difference was no longer significant in the final model. However men who were combining caregiving with part-time work had higher BMIs and waist circumferences which remained in the fully-adjusted models (BMI: 2.42, 95% CI: 0.64, 4.21; waist: 6.33, 95% CI: 2.23, 10.44).

**Table 2:** Associations between informal caregiving and adiposity for UKHLS men.

	Crude association			Age-adjusted			Fully-adjusted^a^		
	BMI	Waist	Body fat	BMI	Waist	Body fat	BMI	Waist	Body fat
	Regression coeff (95% CI)	Regression coeff (95% CI)	Regression coeff (95% CI)	Regression coeff (95% CI)	Regression coeff (95% CI)	Regression coeff (95% CI)	Regression coeff (95% CI)	Regression coeff (95% CI)	Regression coeff (95% CI)
Caregiving									
No	Ref	Ref	Ref	Ref	Ref	Ref	Ref	Ref	Ref
Yes	**1.07** **(0.33, 1.81)**	**3.20** **(1.62, 4.78)**	1.14 (-0.14, 2.41)	0.64 (-0.09, 1.37)	1.21 (-0.31, 2.73)	0.30 (-1.05, 1.65)	0.50 (-0.21, 1.21)	0.86 (-0.65, 2.37)	0.22 (-1.11, 1.54)
R^2^	0.43	0.51	0.15	2.53	8.41	3.33	7.06	13.97	5.45
Caregiving hours									
Not caregiving	**-1.06** **(-1.88, -0.24)**	**-3.17** **(-4.89, -1.45)**	-1.45 (-2.87, -0.02)	-0.65 (-1.46, 0.16)	-1.27 (-2.94, 0.40)	-0.63 (-2.10, 0.84)	-0.55 (-1.35, 0.24)	-1.05 (-2.73, 0.62)	-0.61 (-2.04, 0.82)
1–9 hours/week	Ref	Ref	Ref	Ref	Ref	Ref	Ref	Ref	Ref
10–19 hours/week	-0.37 (-2.47, 1.73)	-2.02 (-6.72, 2.68)	-1.15 (-4.96, 2.65)	-0.36 (-2.34, 1.61)	-1.74 (-5.92, 2.43)	-0.87 (-4.48, 2.74)	-0.35 (-2.27, 1.58)	-1.80 (-5.97, 2.37)	-0.94 (-4.56, 2.69)
20+ hours/week	0.53 (-1.86, 2.92)	2.83 (-1.72, 7.38)	-2.00 (-6.10, 2.10)	0.28 (-2.14, 2.69)	1.53 (-3.22, 6.29)	-2.59 (-6.86, 1.68)	-0.16 (-2.62, 2.31)	0.07 (-4.60, 4.74)	-3.25 (-7.57, 1.07)
R^2^	0.27	0.44	0.26	1.88	8.18	3.32	5.56	13.53	5.32
Number of people cared for									
Not caregiving	**-0.99** **(-1.70, —0.29)**	**-3.36** **(-5.19, -1.53)**	-1.13 (-2.44, 0.17)	-0.55 (-1.24, 0.14)	-1.37 (-3.16, 0.41)	-0.32 (-1.63, 0.99)	-0.43 (-1.11, 0.24)	-1.08 (-2.85, 0.69)	-0.26 (-1.58, 1.06)
1 person	Ref	Ref	Ref	Ref	Ref	Ref	Ref	Ref	Ref
2+ people	0.24 (-1.53, 2.01)	-0.52 (-3.43, 2.39)	0.03 (-3.37, 3.42)	0.27 (-1.47, 2.02)	-0.52 (-3.36, 2.32)	-0.05 (-3.55, 3.45)	0.22 (-1.54, 1.98)	-0.70 (-3.71, 2.30)	-0.14 (-3.61, 3.34)
R^2^	0.25	0.41	0.15	1.87	8.14	3.20	5.55	13.51	5.16
Combining work with caregiving									
Not working or caregiving	0.32 (-0.15, 0.78)	**3.60** **(2.32, 4.87)**	**1.59** **(0.70, 2.49)**	-0.60 (-1.60, 0.40)	-0.05 (-0.70, 0.60)	0.54 (-1.21, 2.29)	0.20 (-0.48, 0.88)	1.20 (-0.56, 2.96)	0.05 (-1.30, 1.41)
Caregiving but not working	0.75 (-0.64, 2.14)	**3.79** **(1.40, 6.19)**	1.55 (-0.79, 3.89)	-0.38 (-1.94, 1.18)	0.17 (-1.22, 1.55)	-0.06 -2.73, 2.61)	0.09 (-1.26, 1.44)	-0.33 (-2.97, 2.31)	-0.57 (-3.31, 2.17)
Caregiving with PT work	**2.88** **(0.85, 4.91)**	**9.05** **(3.70, 14.40)**	3.28 (-2.21, 8.77)	1.83 (-0.30, 3.97)	2.38 (0.39, 4.37)	**6.22** **(1.41, 1.03)**	**2.42** **(0.64, 4.21)**	**6.33** **(2.23, 10.44)**	1.95 (-3.11, 7.01)
Caregiving with FT work	**1.11 (0.25, 1.97)**	**3.93 (1.90, 5.97)**	1.44 (0.03, 2.91)	**-1.54 (-2.65, -0.44)**	0.54 (-0.29, 1.38)	1.36 (-0.58, 3.30)	0.58 (-0.26, 1.42)	1.50 (-0.46, 3.46)	0.51 (-0.98, 1.99)
Working PT but not caregiving	-0.89 (-1.67, -0.11)	-2.03 (-4.35, 0.29)	-0.55 (-2.11,1.00)	-0.55 (-1.38, 0.29)	**-1.00 (-1.78, -0.22)**	**-2.94 (-5.14, -0.75)**	-0.58 (-1.35, 0.18)	-1.62 (3.70, 0.47)	-0.53 (-2.03, 0.97)
Working FT but not caregiving	Ref	Ref	Ref	Ref	Ref	Ref	Ref	Ref	Ref
R^2^	0.89	2.40	0.79	2.91	8.91	3.44	7.34	14.39	5.53
Co-resident caregiving									
Not caregiving	**-0.94 (-1.71, -0.17)**	**-2.70 (-4.38, -1.03)**	-0.98 (-2.17, 0.22)	-0.51 (-1.27, 0.25)	-0.75 (-2.33, 0.82)	-0.16 (-1.37, 1.04)	-0.40 (-1.12, 0.32)	-0.50 (-2.04, 1.04)	-0.14 (-1.35, 1.06)
Non-resident caregiving	Ref	Ref	Ref	Ref	Ref	Ref	Ref	Ref	Ref
Co-resident caregiving	1.15 (-1.43, 3.74)	4.56 (-0.25, 9.37)	1.51 (-4.77, 7.78)	1.15 (-1.45, 3.75)	4.16 (-0.80, 9.12)	1.27 (-5.58, 8.13)	0.91 (-1.99, 3.81)	3.30 (-2.16, 8.75)	0.66 (-5.99, 7.31)
R^2^	0.48	0.63	0.16	2.59	8.50	3.34	7.09	14.0	5.45

Sample size for BMI analyses, n = 3,817; sample size for waist circumference analyses, n = 3,891; sample size for percentage body fat analyses, n = 3, 589

BMI, body mass index; FT, full-time; PT, part-time

^a^Model includes banded age, longstanding health condition, social class, educational attainment, partnership status, number of dependent children, smoking status, and equivalised household income quintiles. Emboldened figures denote statistically significant associations using Bonferroni-corrected p = 0.0167

### Caregiving and adiposity for women

In contrast to men, women who were caregivers had higher adiposity than women who were not caregivers (Table 3, BMI: 1.27, 95% CI: 0.75, 1.79; waist: 2.92, 95% CI: 1.82, 4.02; body fat: 1.90, 95% CI: 1.21, 2.58). An age-caregiving interaction was present for women; it was found that caregiving was associated with particularly high BMI, waist and percentage body fat for younger women (aged 16–44)(Table 4).

**Table 3:** Associations between informal caregiving and adiposity for UKHLS women.

	Crude association			Age-adjusted			Fully-adjusted^a^		
	BMI	Waist	Body fat	BMI	Waist	Body fat	BMI	Waist	Body fat
	Regression coeff (95% CI)	Regression coeff (95% CI)	Regression coeff (95% CI)	Regression coeff (95% CI)	Regression coeff (95% CI)	Regression coeff (95% CI)	Regression coeff (95% CI)	Regression coeff (95% CI)	Regression coeff (95% CI)
Providing informal care									
No	Ref	Ref	Ref	^b^	^b^	^b^	^b^	^b^	^b^
Yes	**1.27** **(0.75, 1.79)**	**2.92** **(1.82, 4.02)**	**1.90** **(1.21, 2.58)**						
R^2^	0.55	0.53	0.66						
Caregiving hours									
Not caregiving	**-0.84** **(-1.38, -0.29)**	**-2.16** **(-3.43, -0.89)**	**-1.62** **(-2.39, -0.85)**	-0.55 (-1.10, -0.01)	-1.23 (-2.50, 0.05)	**-0.95** **(-1.72, -0.17)**	-0.51 (-1.04, 0.03)	-1.12 (-2.38, 0.15)	-0.89 (-1.66, -0.11)
1–9 hours/week	Ref	Ref	Ref	Ref	Ref	Ref	Ref	Ref	Ref
10–19 hours/week	0.81 (-0.63, 2.25)	0.62 (-1.92, 3.16)	**0.36** **(-1.20, 1.91)**	0.79 (-0.65, 2.23)	0.41 (-2.15, 2.98)	0.25 (-1.37, 1.87)	0.62 (-0.84,2.09)	-0.07 (-2.65, 2.51)	-0.01 (-1.62, 1.61)
20+ hours/week	**1.96** **(0.31, 3.61)**	**4.39** **(1.06, 7.72)**	1.48 (-0.30, 3.26)	1.81 (0.18, 3.44)	3.83 (0.57, 7.08)	1.21 (-0.55, 2.96)	1.29 (-0.29,2.88)	2.54 (-0.59, 5.68)	0.55 (-1.12, 2.23)
R^2^	0.75	0.71	0.72	2.64	5.43	6.24	6.70	10.85	9.04
Number of people cared for									
Not caregiving	**-0.74** **(-1.24, -0.23)**	**-1.94** **(-3.11, -0.76)**	**-1.49** **(-2.24, -0.73)**	-0.48 (-1.00, 0.04)	-1.08 (-2.27, 0.11)	-0.87 (-1.64, -0.10)	-0.38 (-0.90, 0.14)	-0.88 (-2.07, 0.32)	-0.74 (-1.50, 0.03)
1 person	Ref	Ref	Ref	Ref	Ref	Ref	Ref	Ref	Ref
2+ people	**1.71** **(0.52, 2.89)**	**3.14** **(0.73, 5.55)**	1.34 (0.01, 2.66)	**1.54** **(0.35, 2.73)**	2.54 (0.08, 4.99)	0.98 (-0.42, 2.37)	1.35 (0.18, 2.53)	1.95 (-0.43, 4.33)	0.76 (-0.62, 2.13)
R^2^	0.80	0.69	0.74	2.67	5.77	6.25	7.00	10.84	9.69
Combining work with caregiving									
Not working or caregiving	0.28–0.27, 0.84)	**2.39 (1.16, 3.63)**	**1.04 (0.21, 1.88)**	^b^	^b^	^b^	^b^	^b^	^b^
Caregiving but not working	**1.43 (0.55, 2.31)**	**4.97 (3.12, 6.82)**	**2.78 (1.66, 3.90)**						
Caregiving with PT work	0.94 (-0.07, 1.96)	2.27 (0.22, 4.32)	**1.77 (0.58, 2.95)**						
Caregiving with FT work	**1.36 (0.40, 2.31)**	**3.64 (1.49, 5.79)**	**2.40 (1.08, 3.73)**						
Working PT but not caregiving	-0.44 (-0.98, 0.10)	-0.45 (-1.71, 0.81)	0.15 (-0.68, 0.99)						
Working FT but not caregiving	Ref	Ref	Ref						
R^2^	0.78	1.36	0.93						
Co-resident caregiving									
Not caregiving	**-1.11 (-1.63, -0.59)**	**-2.59 (-3.69, -1.48)**	**-1.78 (-2.47, -1.09)**	**-0.81 (-1.34, -0.28)**	**-1.60 (-2.72, -0.48)**	**-1.07 (-1.77, -0.37)**	**-0.71 (-1.24, -0.18)**	**-1.39 (-2.52, -0.26)**	**-0.93 (-1.63, -0.23)**
Non-resident caregiving	Ref	Ref	Ref	Ref	Ref	Ref	Ref	Ref	Ref
Co-resident caregiving	-1.49 (-0.53, 3.50)	3.08 (-0.86, 7.01)	1.08 (-1.07, 3.23)	1.35 (-0.61, 3.31)	2.47 (-1.42, 6.35)	0.89 (-1.28, 3.06)	0.82 (-1.08, 2.73)	0.86 (-2.86, 4.58)	0.31 (-1.78, 2.39)
R^2^	0.64		0.68	2.54	5.71	6.22	6.87	10.79	9.67

Sample size for BMI analyses, n = 5 373; sample size for waist circumference analyses, n = 5 427; sample size for percentage body fat analyses, n = 5 189BMI–body mass index; FT–full-time; PT–part-time

^a^Model includes banded age, longstanding health condition, social class, educational attainment, partnership status, number of dependent children, smoking status, and equivalised household income quintiles. Emboldened figures denote statistically significant associations using Bonferroni-corrected p = 0.0167

^b^Age- and fully-adjusted models not shown as age interactions were statistically significant. Age-stratified associations shown in Table 4.

**Table 4:** Associations between informal caregiving, combining caregiving and work with adiposity for UKHLS women stratified by age group.

BMI	Crude association			Fully-adjusted^a^		
	16–44 yrs	45–64 yrs	65+ yrs	16–44 yrs	45–64 yrs	65+ yrs
Providing informal care						
No	Ref	Ref	Ref	Ref	Ref	Ref
Yes	**1.94 (1.04, 2.85)**	0.63 (-0.10, 1.35)	-0.42 (-1.57, 0.72)	**1.42 (0.49, 2.35)**	0.60 (-0.12, 1.32)	-0.08 (-1.31, 1.16)
R^2^	0.88	0.18	0.08	5.46	6.23	8.80
Combining work with caregiving						
Not working or caregiving	-0.23 (-1.14, 0.67)	-0.05 (-0.95, 0.86)	1.69 (-1.55, 4.91)	**-1.34 (-2.24, -0.44)**	**-1.30 (-2.28, -0.32)**	-0.31 (-3.57, 2.95)
Caregiving but not working	**2.21 (0.67, 3.75)**	0.53 (-0.94, 1.99)	1.20 (-2.32, 4.73)	0.69 (-0.87, 2.25)	-0.59 (-2.03, 0.85)	-0.46 (-3.99,3.07)
Caregiving with PT work	1.07 (-0.37, 2.51)	0.08 (-1.12, 1.29)	0.79 (-2.84, 4.41)	-0.69 (-2.23, 0.85)	-0.49 (-1.64, 0.66)	-0.22 (-3.74, 3.29)
Caregiving with FT work	**2.37 (0.49, 4.26)**	-0.13 (-1.37, 1.10)	**7.28 (4.06, 10.51)**	**2.07 (0.16, 3.99)**	-0.17 (-1.38, 1.03)	**7.06 (3.38, 10.74)**
Working PT but not caregiving	-0.08 (-0.84, 0.68)	**-1.29 (-2.15, -0.43)**	1.52 (-1.68, 4.71)	-1.04 (-1.90, -0.18)	**-1.88 (-2.74, -1.03)**	0.02 (-3.53, 3.57)
Working FT but not caregiving	Ref	Ref	Ref	Ref	Ref	Ref
R^2^	0.99	1.03	0.44	6.33	7.49	9.12
**Waist**	**Crude association**			**Fully-adjusted**^**a**^		
	**16–44 yrs**	**45–64 yrs**	**65+ yrs**	**16–44 yrs**	**45–64 yrs**	**65+ yrs**
Providing informal care						
No	Ref	Ref	Ref	Ref	Ref	Ref
Yes	**4.29 (2.31, 6.27)**	1.35 (-0.28, 2.98)	-2.53 (-5.58, 0.51)	**2.93 (0.86, 5.00)**	1.28 (-0.35, 2.90)	-2.21 (-5.45, 1.04)
R^2^	0.83	0.16	0.38	7.19	6.36	8.52
Combining work with caregiving						
Not working or caregiving	0.64 (-1.34, 2.62)	0.13 (-1.95, 2.21)	3.97 (-6.24, 14.19)	-1.88 (-3.80, 0.04)	-2.43 (-4.62, -0.23)	0.25 (-9.76, 10.25)
Caregiving but not working	**6.78 (3.70, 9.87)**	2.34 (-1.04 5.71)	0.71 (-9.75, 11.18)	3.23 (0.03, 6.44)	-0.03 (-3.40, 3.33)	-2.81 (-13.19, 7.57)
Caregiving with PT work	2.28 (-1.18, 5.74)	-0.62 (-3.31, 2.08)	3.76 (-6.74, 14.26)	-1.99 (-5.65, 1.67)	-1.68 (-4.25, 0.88)	2.42 (-8.51, 13.34)
Caregiving with FT work	**4.68 (0.98, 8.38)**	-0.14 (-3.00, 2.72)	**34.27 (24.17, 44.38)**	**4.01 (0.21, 7.80)**	-0.28 (-3.04, 2.47)	**34.09 (22.82, 45.36)**
Working PT but not caregiving	0.25 (-1.44, 1.94)	**-2.53 (-4.49, -0.57)**	3.16 (-8.62, 14.94)	-2.09 (-3.99, -0.18)	**-3.68 (-5.65, -1.71)**	0.48 (-10.88, 11.85)
Working FT but not caregiving	Ref	Ref	Ref	Ref	Ref	Ref
R^2^	1.05	0.98	1.68	7.86	7.32	9.87
**Body fat**	**Crude association**			**Fully-adjusted**^**a**^		
	16–44 yrs	**45–64 yrs**	**65+ yrs**	**16–44 yrs**	**45–64 yrs**	**65+ yrs**
Providing informal care						
No	Ref	Ref	Ref	Ref	Ref	Ref
Yes	**3.11 (1.80, 4.42)**	0.31 (-0.72, 1.33)	-0.74 (-2.06, 0.59)	**2.48 (1.16, 3.80)**	0.25 (-0.75, 1.25)	-0.71 (-2.06, 0.63)
R^2^	1.11	0.03	0.14	6.46	5.18	3.85
Combining work with caregiving						
Not working or caregiving	-0.002 (-1.35, 1.35)	-0.11 (-1.23, 1.02)	2.80 (-4.72, 10.32)	-1.51 (-2.95, -0.07)	-1.24 (-2.43, -0.04)	2.40 (-5.35, 10.14)
Caregiving but not working	**3.73 (1.56, 5.89)**	0.37 (-1.61, 2.36)	2.13 (-5.70, 9.97)	1.71 (-0.55, 3.97)	-0.65 (-2.59, 1.29)	1.71 (-6.35, 9.77)
Caregiving with PT work	2.18 (0.04, 4.33)	-0.35 (-1.95, 1.23)	1.30 (-6.98, 9.59)	-0.03 (-2.21, 2.16)	-0.70 (-2.26, 0.85)	1.10 (-7.24, 9.44)
Caregiving with FT work	**4.08 (1.42, 6.74)**	-0.49 (-2.10, 1.12)	4.57 (-3.01, 12.15)	**3.67 (1.00, 6.35)**	-0.62 (-2.19, 0.95)	6.17 (-2.29, 14.64)
Working PT but not caregiving	0.58 (-0.60, 1.76)	-1.25 (-2.36, -0.14)	3.69 (-4.18, 11.56)	-0.80 (-2.07, 0.46)	**-1.61 (-2.77, -0.44)**	3.31 (-4.71, 11.32)
Working FT but not caregiving	Ref	Ref	Ref	Ref	Ref	Ref
R^2^	1.29	0.56	0.46	7.06	5.81	4.14

Sample size for BMI analyses, n = 5 707; sample size for waist circumference analyses, n = 5 764; sample size for percentage body fat analyses, n = 5 508

^a^Model includes banded age, longstanding health condition, social class, educational attainment, partnership status, number of dependent children, smoking status, and equivalised household income quintiles. Emboldened figures denote statistically significant associations using Bonferroni-corrected p = 0.0167

Women who provided more hours of caregiving (>20 hours per week), had mean BMIs which were almost two points higher (1.96, 95% CI: 0.31, 3.61) and waists that were, on average, more than four centimetres larger (4.39cm, 95% CI: 1.06cm, 7.72cm). However upon inclusion of age, these associations were no longer statistically significant according to the Bonferroni corrected p-value. Also, women who were caring for at least two people had higher adiposity but these associations no longer remained after inclusion of all covariates. No association between residential or non-residential caregiving and adiposity was observed.

Compared to women who were working full-time and not caregiving, women who were working full-time combined with caregiving had higher levels of adiposity as indicated by all three outcome measures. A significant age-caregiving and work interaction was found. The age-stratified associations presented in Table 4 suggest that this was found to be driven by particularly high levels of adiposity amongst younger (16–44 years) and older (65+ years) women who were combining caregiving with full-time work. For instance, women aged 16–44 years combining full-time work with caregiving had BMIs which were more than two points higher (2.07, 95% CI: 0.16, 3.99) and women aged 65 or over had BMIs more than seven points higher (7.06, 95% CI: 3.38, 10.74). The increased adiposity of younger and older women combining full-time work with caregiving was even more pronounced for waist circumference (16–44 years, waist: 4.01, 95% CI: 0.21, 7.80; 65+ years, waist: 34.09, 95% CI: 22.82, 45.36). Women aged 16–44 years combining full-time work with caregiving also had higher percentage body fat.

## Discussion

Using UK longitudinal data we found that informal caregiving was associated with higher adiposity for women but not men. We found that the association between informal caregiving and adiposity was driven by younger women aged 16–44 showing particularly strong associations with adiposity. Secondly we found that the combination of paid work and caregiving was important for men and women. Men combining part-time work and caregiving had higher adiposity, as did women who were combining full-time work and caregiving, particularly for younger (aged 16–44) and older (aged 65+) women.

Whilst women might be more likely to adopt a caregiving role [24], their health may be impacted upon more negatively than men’s. Indeed previous research showed that women caregivers had poorer health than men caregivers [8,26,27,31,32]. The gender differences observed may be because women are more likely to be primary caregivers [33] and therefore may experience greater burden and responsibility for the care recipient. Women are also more likely to adopt more intensive caregiving activities, including more hours and the provision of personal care and assistance with activities of daily living [34], the latter of which we were unable to investigate here. Interestingly, we found no association between caregiving hours and adiposity for men or women. This is in contrast to previous research using the UK Census to investigate associations between caregiving and caregiving intensity with self-rated health [20], where the greater the hours of care provided, the greater the negative impact upon health.

We extend previous work, which has largely focused on psychological health, to adiposity. One previous study investigated the relationship between caregiving and weight gain [12] and found that caregiving was associated with increased BMI and obesity, particularly for men. Whilst, for men, this is inconsistent with our findings, this study was based upon a small sample (n = 167) of caregivers to a specific patient group (Alzheimer’s disease) which may not be generalisable to a broader population. Other work by Hajek and colleagues [17,35] found that men in the German Aging Study who were informal caregivers had increased BMI. This association was not observed for women. Again the discrepancy in findings might come from differences in the study sample, as the present study is a wider population of adults aged 16+.

We additionally found that younger women caregivers had particularly higher levels of adiposity. This may be due to caregiving outside of the normative life stage, possibly combined with work and other family responsibilities, such as the raising of children. Indeed we found that women caregivers who were combining full-time paid work and caregiving responsibilities had higher adiposity, and this was again particularly the case for younger (aged 16–44) and older (aged 65+) women in our sample. We also found that combining part-time paid work and caregiving was associated with increased adiposity for men. Younger carers may also experience a particularly greater loss in social support, especially as caregiving is a non-normative experience during this life stage. Our study is one of the first to investigate caregiving for adults aged 16+, rather than restricting our analyses to mid-life onwards.

Our findings also suggest that it is important to consider caregiving characteristics and the combination with other social roles. For instance, as mentioned above, we found that caregiving status was not associated with adiposity for men, but the combination of part-time working and caregiving was. Informal caring is time consuming and relatively inflexible and therefore places time constraints on paid work [27]. Previous research using the UKHLS found that women caregivers were much more likely to leave part-time (OR = 2.64, 95% CI: 1.46, 4.79) or almost full-time work (OR = 4.46, 95% CI: 2.53, 7.88), compared to women who were not caregivers [21]. Time constraints also affect the quantity of leisure time available and the time available for preparing healthy, nutritious meals and undertaking physical exercise. Indeed a German study found that informal caregivers had lower participation in sporting activities compared to non-caregiving peers [35]. Additionally, psychosocial stress can directly affect fat deposition processes, and this might be another mechanism through which informal caregiving might result in higher adiposity [16]. Whilst our initial hypothesis was that informal caregiving is likely to be associated with increased adiposity and weight gain, we also acknowledge that psychosocial stress and any physical exertion required might result in weight loss and a reduction in adiposity for some people. Further exploration of our data showed that informal caregivers were not more likely to be underweight as well, although only 27 men and 61 women in our sample were classified as underweight using the World Health Organisation’s classification of obesity [36].

### Strengths and limitations

Our study has a few limitations. Firstly, we used complete case analysis. Those with complete data and missing data (Table 1) differed on most characteristics; our sample was more socially advantaged and less likely to be caregiving. Consequently, if we were able to analyse the full sample we’d include more socially disadvantaged people, including more caregiving and people with higher levels of adiposity. Therefore we expect our findings to be an underestimation of the associations seen, had the full data been utilised. Secondly, we only had adiposity data at one wave and this was early in the UKHLS. We were therefore unable to look at adiposity change, control for adiposity prior to caregiving, or to investigate longer-term associations between caregiving and adiposity. We were also unable to rule out the possibility that adiposity preceded informal caregiving. Thirdly, we were unable to explore caregiving duration, caregiving strain, the relationship between the caregiver and care recipient, the reasons the care recipient required care or the specific caregiving activities undertaken. These aspects of caregiving are likely to be important for health. Further research is therefore needed with a different dataset to explore these aspects of caregiving. Despite these limitations our study also had a number of strengths. We used a large, longitudinal UK dataset which allowed us to control for the caregivers’ health status at baseline, which would not be possible using a cross-sectional study. Unlike much previous research, this was a general population sample and included people aged 16 and over. Our findings about the heightened risk of younger women caregivers is therefore an important addition to the evidence base on caregiving and health. Finally, BMI, waist circumference and percentage body fat were measured by trained study nurses.

In summary, we found that informal caregiving was associated with higher adiposity, particularly for younger women. The combination of caregiving and paid work responsibilities also appears to be important for adiposity for both men and women. Further research is required to investigate potential mechanisms, including caregiver burden, loss of social support, depression, financial strain, and risky health behaviours. Given the increasing prevalence of informal caregiving and its societal and financial importance, caregiver health should be a public health priority.
